# Alternative Splicing of *CARMA2/CARD14* Transcripts Generates Protein Variants With Differential Effect on NF-κB Activation and Endoplasmic Reticulum Stress-Induced Cell Death

**DOI:** 10.1002/jcp.22667

**Published:** 2011-02-02

**Authors:** Ivan Scudiero, Tiziana Zotti, Angela Ferravante, Mariangela Vessichelli, Pasquale Vito, Romania Stilo

**Affiliations:** 1 BiogemAriano Irpino, Italy; 2 Dipartimento di Scienze Biologiche ed Ambientali, Università degli Studi del SannioBenevento, Italy

## Abstract

The caspase recruitment domain (CARD)-containing proteins CARMA1-3 share high degree of sequence, structure and functional homology. Whereas CARMA1 and CARMA3 have been identified as crucial components of signal transduction pathways that lead to activation of NF-κB transcription factor, little is known about the function of CARMA2. Here we report the identification of two splice variants of CARMA2. One transcript, named CARMA2*short* (CARMA2*sh*), is predicted to encode for a CARMA2 polypeptide containing the CARD, coiled coil, and a PDZ domains, but lacking the SH3 and the GuK domains. The second variant, CARMA2*cardless* (CARMA2*cl*), encodes for a polypeptide lacking the CARD domain and containing only a portion of the coiled coil domain and a linker region. Expression analysis confirmed the presence of the CARMA2 alternatively spliced transcripts in both human cell lines and tissues. Fluorescence microscopy data show that both splice variants localize in the cytosol. Biochemical experiments indicate that CARMA2*sh* interacts with TRAF2 and activates NF-κB in a TRAF2-dependent manner. Finally, CARMA2*sh* variant protects cells from apoptosis induced by different stimuli. Taken together, these results demonstrate that multiple transcripts encoding several CARMA2 isoforms exist in vivo and regulate NF-κB activation and apoptosis. J. Cell. Physiol. 226: 3121–3131, 2011. © 2011 Wiley Periodicals, Inc.

The caspase recruitment domain (CARD)-containing proteins CARMA1 (CARD11/Bimp2) (Bertin et al., 2001; Gaide et al., 2001; Wang et al., 2001), CARMA2 (CARD14/Bimp3) (Bertin et al., 2001), and CARMA3 (CARD10/Bimp1) (Wang et al., 2001; McAllister-Lucas et al., 2001) share a high degree of sequence, structure, and functional homology. CARMA proteins belong to the membrane-associated guanylate kinase (MAGUK) family of proteins, which can function as molecular scaffolds that assist recruitment and assembly of signal transduction molecules (Dimitratos et al., 1999). CARMA proteins contain a CARD domain, a Src-homology 3 (SH3) domain, one or several PDZ domains and a GuK domain (Bertin et al., 2001; Gaide et al., 2001; Wang et al., 2001; McAllister-Lucas et al., 2001). Genetic and biochemical studies have identified CARMA1 as crucial component of a complex of proteins that links antigen receptors on B and T lymphocytes to activation of NF-κB (Wang et al., 2002; Gaide et al., 2002; Pomerantz et al., 2002; Hara et al., 2003; Egawa et al., 2003; Newton and Dixit, 2003; Jun et al., 2003). This complex, which includes CARMA1, BCL10, MALT1, and TRAF6, has been shown to activate the IKK complex through an ubiquitylation-dependent pathway (Schulze-Luehrmann and Ghosh, 2006).

A second member of the CARMA family, CARMA3, is required for activation of NF-κB induced by G-protein-coupled receptors (GPCRs) (Grabiner et al., 2007; McAllister-Lucas et al., 2007). In fact, it has been demonstrated that a complex of proteins, including CARMA3, BCL10, and MALT1, appears to be responsible for transmitting the signal from the GPCRs to the IKK complex (Grabiner et al., 2007; McAllister-Lucas et al., 2007). By contrast, although overexpression of CARMA2 also induces NF-κB activation in HEK-293 cells (Bertin et al., 2001), the signaling pathway(s) regulated by this protein is still unknown. Here we describe the identification of two splice variants of CARMA2 and show data indicating an involvement of these proteins in regulation of NF-κB activation and endoplasmic reticulum (ER)-stress induced cell death.

## Results

Previous work identified CARMA2/CARD14 (CARMA2FL) as a 1,004 amino acidic residues protein belonging to the CARMA family of proteins (Bertin et al., 2001). To examine the occurrence of splice variants derived from the human *CARMA2* gene, we performed an extensive reverse transcription-polymerase chain reaction (RT-PCR) analysis of mRNAs isolated from human cell lines using *CARMA2/CARD14* exon-specific primers.

**Fig. 1:**
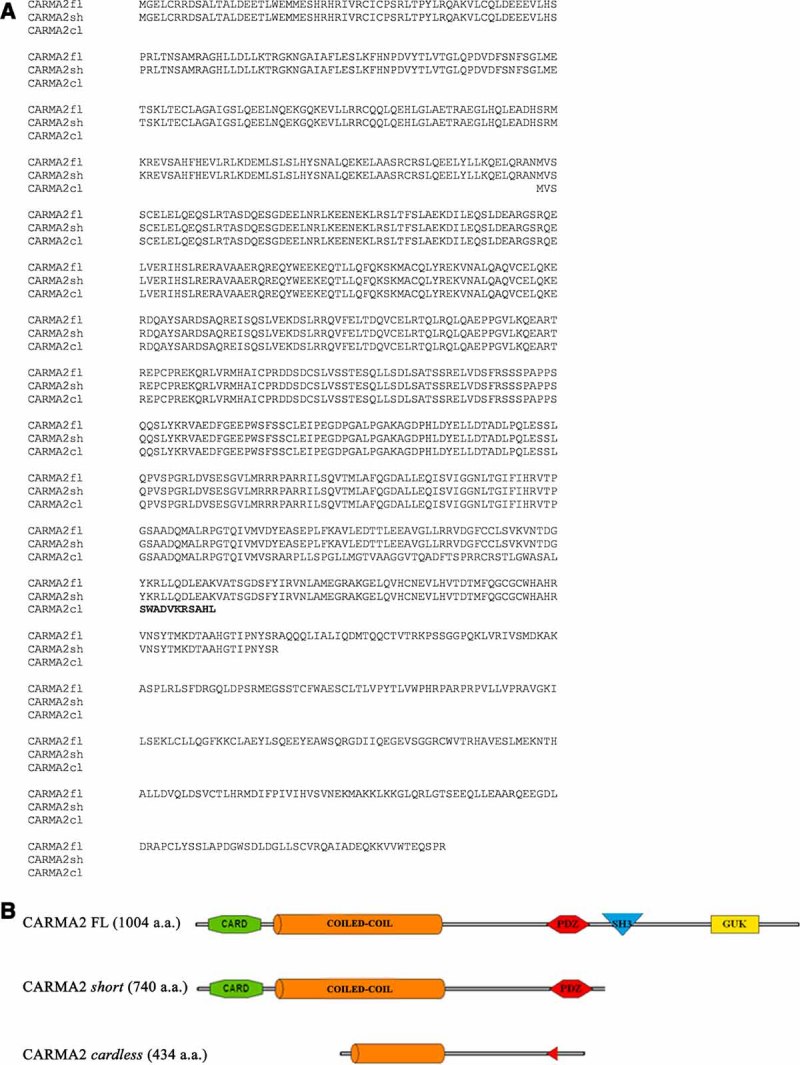
*CARMA2* isoforms. A: Amino acid alignment of CARMA2FL, CARMA2*sh*, and CARMA2*cl*. The unique sequence of CARMA2*cl* is in bold. B: Schematic representation of CARMA2FL, CARMA2*sh*, and CARMA2*cl* isoforms.

Such analysis revealed the existence of two different splice variants of *CARMA2/CARD14*. One variant contains the same translational start of CARMA2FL but includes an additional exon (*alternative exon 15*) containing an alternative stop codon. This transcript is predicted to encode for a polypeptide corresponding to amino acids 1–740 of CARMA2FL, and therefore we named this variant CARMA2*short* (CARMA2*sh*) (Fig. 1A,B). The second variant mRNA skips exons 1–4, contains an alternative translational start codon in exon 5 and includes an additional exon containing an alternative stop codon (Fig. 1A,B). The *CARMA2* isoform encoded by this splice variant would lack the CARD domain, includes amino acids Met^238^-Val^618^ of CARMA2FL and contains a unique carboxy terminus (Fig. 1A,B). We named this variant CARMA2*cardless* (CARMA2*cl*).

**Fig. 2:**
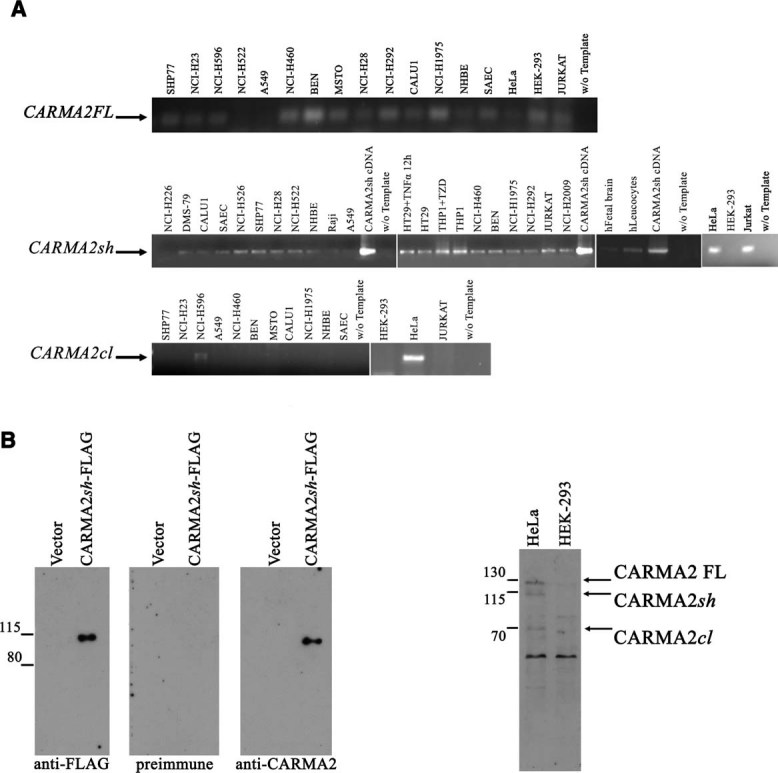
*CARMA2* isoform expression. A: RT-PCR was used to analyze CARMA2 transcripts in a part of cDNA from various human tissues and cell lines. These comprised human lung carcinoma (NCI-H226, DMS-79, CALU1, NCI-H526, SHP77, A549, NCI-H460, NCI-H1975, NCI-H292, and NCI-H2009) mesothelioma (NCI-H28) normal epithelial (SAEC, NHBE), Burkitt's limphoma (Raji), colon carcinoma (HT29), monocitic leukemia (THP1), bronchial carcinoma (BEN), T cell leukemia (Jurkat), and cervical carcinoma (HeLa). Where indicated, cells were stimulated with tumor necrosis factor α (TNFα) or thiazolidinedione (TZD) before RNA extraction. Identity of amplicons was confirmed by DNA sequencing. B: Specificity of the antiserum to CARMA2 used in this study. HEK-293 cells were transfected with a vector that was empty or expressing FLAG-tagged CARMA2*sh*. Twenty-four hours later, cell lysates were separated by SDS–PAGE and transferred onto membranes subsequently probed with anti-FLAG mAb, affinity-purified anti-CARMA2 rabbit antiserum or preimmune rabbit serum, as indicated. C: Cell lysates were prepared from HeLa and HEK-293 cells, separated by SDS–PAGE, and transferred onto membranes subsequently probed with anti-CARMA2 antisera.

To confirm the existence of these alternatively spliced transcripts of *CARMA2*, we performed RT-PCR on mRNAs isolated from different human normal and tumoral cell lines and normal tissues using internal primers that can distinguish each of the splice variants. Using a primer set specific for exon 15 and the alternative exon 15, we detected a PCR product that corresponds to the predicted size of a fragment derived from transcripts encoding CARMA2*sh* (Fig. 2A). This transcript was expressed in most of the cell lines tested, in human fetal brain and human leucocytes (Fig. 2A).

We also confirmed the presence of the transcript coding for CARMA2*cl* using a primer within the 5′-untranslated region paired with a reverse primer located in exon 5. The CARMA2*cl* transcript was expressed only in HeLa cells (Fig. 2A).

To study these CARMA2 splice variants, we generated a polyclonal rabbit antibody directed against a polypeptide corresponding to aa 108–407 of CARMA2 FL (Fig. 2B). When probed on cell lysates prepared from HeLa and HEK-293 cells, the antisera detected bands corresponding to the predicted size of endogenous CARMA2FL, CARMA2*sh*, and CARMA2*cl* isoforms. The antisera also recognizes additional bands, indicating that other splice variants of CARMA2 may occur (Fig. 2C).

**Fig. 3:**
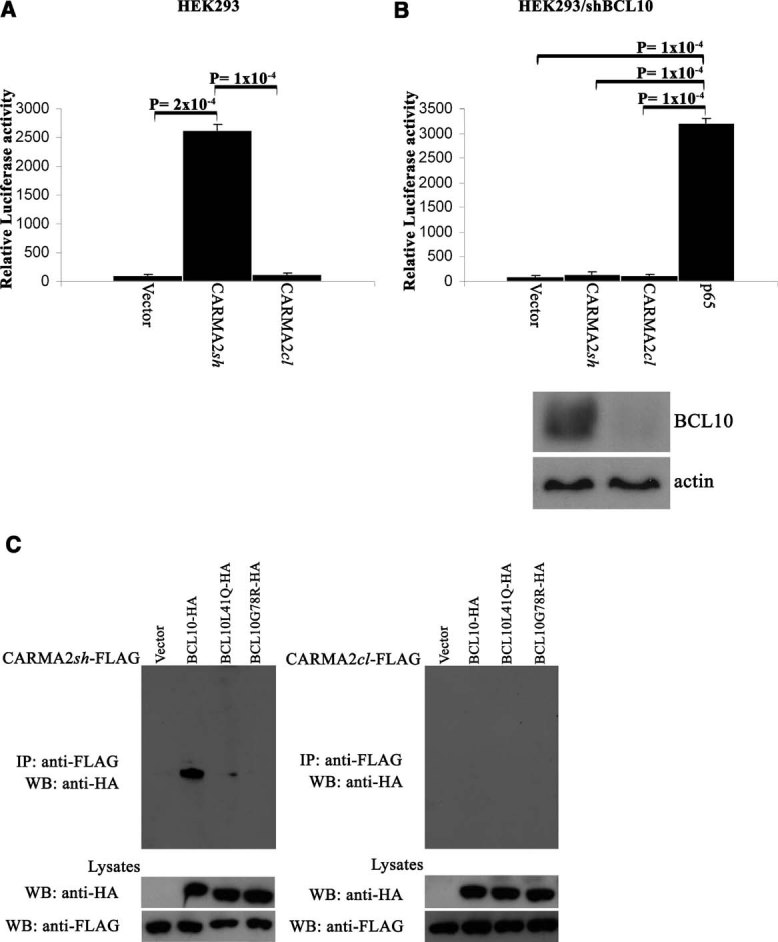
CARMA2*sh* interacts with BCL10. A: HEK-293 cells were transiently cotransfected with an expression vector encoding for the indicated polypeptides, together with pNF-κB-luc and pRSV-βgal reporter vectors. The total amount of transfected plasmidic DNA was maintained constant by adding empty vector. Sixteen hours after transfection, cell lysates were prepared and luciferase activity was measured. Data shown represent relative luciferase activity normalized on β-galactosidase activity and is representative of six independent experiments done in triplicate. Statistical analysis was by the one-tailed unpaired Student's *t*-test. B: The indicated expression vectors were transfected as in (A) in HEK-293 cells expressing a shRNA targeting BCL10. Expression of BCL10 was monitored by immunoblot assay (bottom part). Statistical analysis was by the one-tailed unpaired Student's *t*-test. C: HEK-293 cells were transiently cotransfected with a FLAG-tagged version of CARMA2*sh* (left parts) or CARMA2*cl* (right parts) along with HA-tagged version of wt and point mutants of BCL10, as indicated. Twenty-four hours later, cell lysates were immunoprecipitated with anti-FLAG mAb. Immunocomplexes were separated by SDS–PAGE and transferred onto membranes subsequently probed with anti-HA antisera.

Because CARMA proteins are implicated in NF-κB signaling pathways, we first determined whether CARMA2*sh* and CARMA*cl* can induce NF-κB activity using a luciferase reporter assay. When CARMA2*sh* was expressed in HEK-293 cells, NF-κB activity was induced at least 20- to 40-fold compared with empty vector (Fig. 3A). In the same assay, CARMA*cl* was unable to promote NF-κB activity, thus confirming that the N-terminal CARD domain of CARMA proteins is essential for NF-κB signaling (Fig. 3A).

It has been shown that CARMA3, a member of the CARMA family of proteins, is unable to activate NF-κB in the absence of functional BCL10 (Wang et al., 2001). To test whether also CARMA2*sh* requires BCL10 for the NF-κB-inducing activity, CARMA2*sh* was tested in HEK-293 cells expressing a short hairpin RNA (shRNA) designed to target BCL10 for degradation by the RNAi pathway. The results of these experiments indicate that, likewise CARMA3, CARMA2*sh* as well depends on functional BCL10 to induce NF-κB (Fig. 3B). Similarly to CARMA1 and CARMA3 (Stilo et al., 2008), activation of NF-κB mediated by CARMA2*sh* was inhibited by expression of A20 (data not shown).

Biochemical data confirmed our functional observation. In fact, to verify whether CARMA2 splice variants bind to BCL10, CARMA2*sh*, and CARMA2*cl* were co-expressed with BCL10, cell extracts were prepared, and were immunoprecipitated with anti-FLAG antibody. Immunoblotting experiments revealed that CARMA2*sh*, but not CARMA2*cl*, co-immunoprecipitated with BCL10 (Fig. 3C). The binding of CARMA2*sh* to BCL10 was dependent on an intact CARD of BCL10, because CARMA2*sh* failed to co-precipitate variants of BCL10 containing a point mutation L41Q or G78R that disrupts CARD/CARD interactions (Srinivasula et al., 1999; Guiet and Vito, 2000) (Fig. 3C). These results indicate that CARMA2*sh*, but not CARMA2*cl*, associates with BCL10 through a CARD/CARD homotypic interaction.

**Fig. 4:**
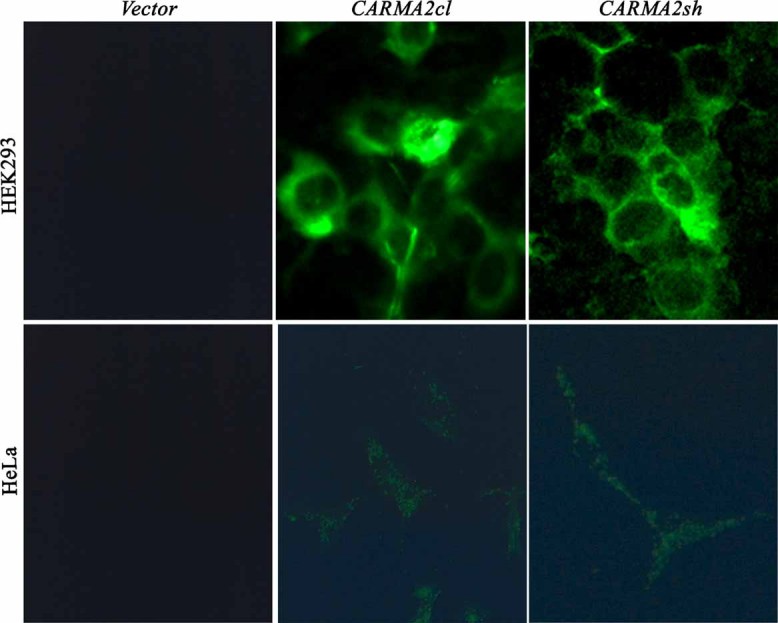
Subcellular localization of *CARMA2* isoforms. HEK-293 (upper parts) and HeLa cells (lower parts) were transfected with mammalian FLAG-tagged vector, empty (vector) or expressing CARMA2*sh* or CARMA2*cl*. Sixteen hours after transfection, cells were stained with anti-FLAG mAb, followed by FITC-conjugated anti-mouse IgG.

We next determined the cellular localization of CARMA2*sh* and CARMA2*cl*. For this, we transfected HEK-293 cells with a FLAG-tagged vector encoding for these splice variants of CARMA2, and the expressed protein was detected using a monoclonal anti-FLAG antibody. The results of these experiments, shown in Figure 4, indicate that both CARMA2*sh* and CARMA2*cl* exhibit a clear pattern of diffuse cytoplasmic distribution.

**Fig. 5:**
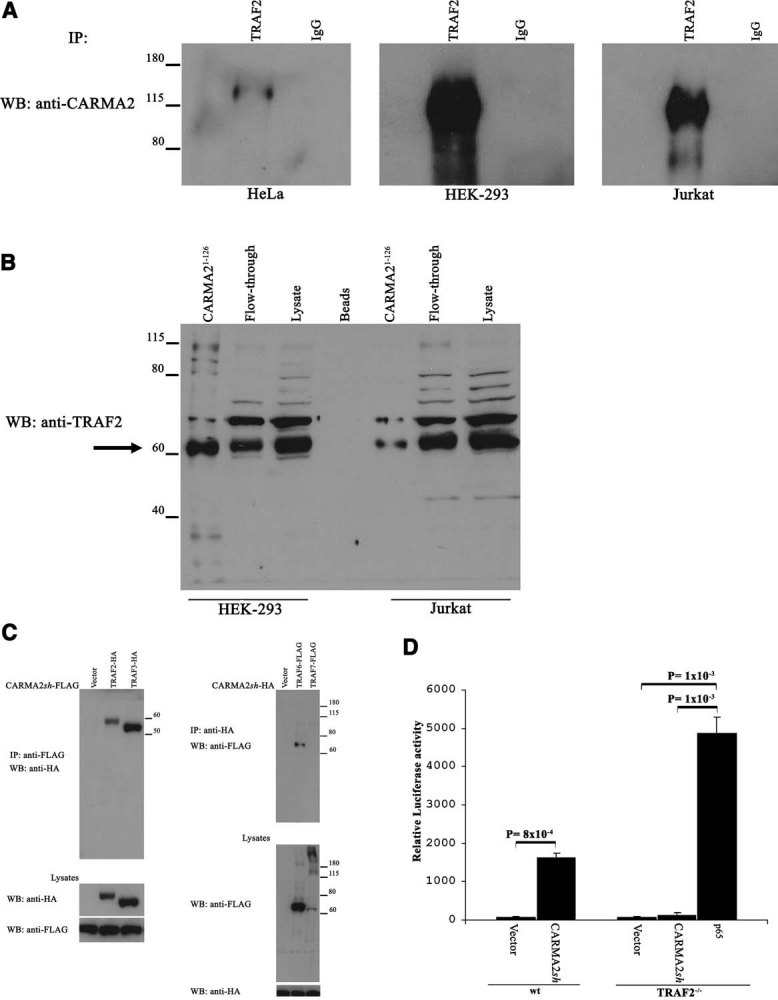
CARMA2*sh* binds to and activates NF-κB through TRAF2. A: Lysates prepared from HeLa, HEK-293, or Jurkat cells were immunoprecipitated with anti-TRAF2 antibody or an isotype-matched antibody. Immunoprecipitating material was separated by SDS–PAGE and blotted onto membranes subsequently hybridized with anti-CARMA2 rabbit antiserum. B: Recombinant histidine-tagged CARMA2^1–126^ was purified with nickel–nitrilotriacetic acid-agarose beads and mixed with lysates from HEK-293 or Jurkat cells. After washing, agarose beads were boiled in SDS-sample buffer, separated by SDS–PAGE, and transferred onto nitrocellulose membrane probed with anti-TRAF2 antibody. C: HEK-293 cells were transiently cotransfected with a tagged version of CARMA2*sh* along with tagged version of members of TRAF proteins. Twenty-four hours later, cell lysates were immunoprecipitated with anti-FLAG mAb or anti-HA antisera. Immunocomplexes were separated by SDS–PAGE and transferred onto membranes subsequently probed with anti-HA antisera or anti-FLAG mAb. D: CARMA2*sh* fails to activate NF-κB in TRAF2-deficient cells wt MEFs (left columns) and TRAF2^−/−^ MEFs (right columns) were transfected with pNF-κB-Luc, pCMV-β-gal, and the indicated expression plasmids. An expression vector coding for the NF-κB subunit p65 served as positive control. Cells were harvested 24 h after transfection, and NF-κB activity was determined. Data shown represents relative luciferase activity normalized on β-galactosidase activity and is representative of four independent experiments done in triplicate. Statistical analysis was by the one-tailed unpaired Student's *t*-test.

Interestingly, in a set of association experiments, we also found that immunocomplexes containing endogenous TRAF2, a scaffold protein that transduces signals from membrane receptors and the ER membrane (Rothe et al., 1994; Yoneda et al., 2001; Mauro et al., 2006), also contain CARMA2 isoforms (Fig. 5A). To confirm association of CARMA2*sh* and TRAF2 in a different experimental system, a polypeptide corresponding to Met^1^-Glu^126^ of CARMA2FL was expressed as His-tagged protein in bacteria and tested for binding to TRAF2 endogenously expressed. The results of these pull-down assays, shown in Figure 5B, indicate that recombinant CARMA2 Met^1^-Glu^126^ binds to TRAF2 in lysates prepared from HEK-293 and Jurkat cells. Similar results were observed when lysates were prepared from HeLa cell (data not shown). In addition, coprecipitation experiments demonstrated that CARMA2*sh* also binds to TRAF3, TRAF6 but not TRAF7 (Fig. 5C). To further define the biological significance of CARMA2*sh* interaction with TRAF2, we tested the NF-κB inducing activity of CARMA2*sh* in embryonic fibroblasts (MEFs) derived from TRAF2^−/−^ mice and wt MEFs. As shown in Figure 5D, when CARMA2*sh* was expressed in wt MEFs, NF-κB was induced 10- to 15-fold compared with empty vector. However, CARMA2*sh* was barely able to induce NF-κB signaling in TRAF2^−/−^ MEFs. Thus, a functional TRAF2 protein is required for activation of NF-κB induced by CARMA2*sh* expression.

**Fig. 6:**
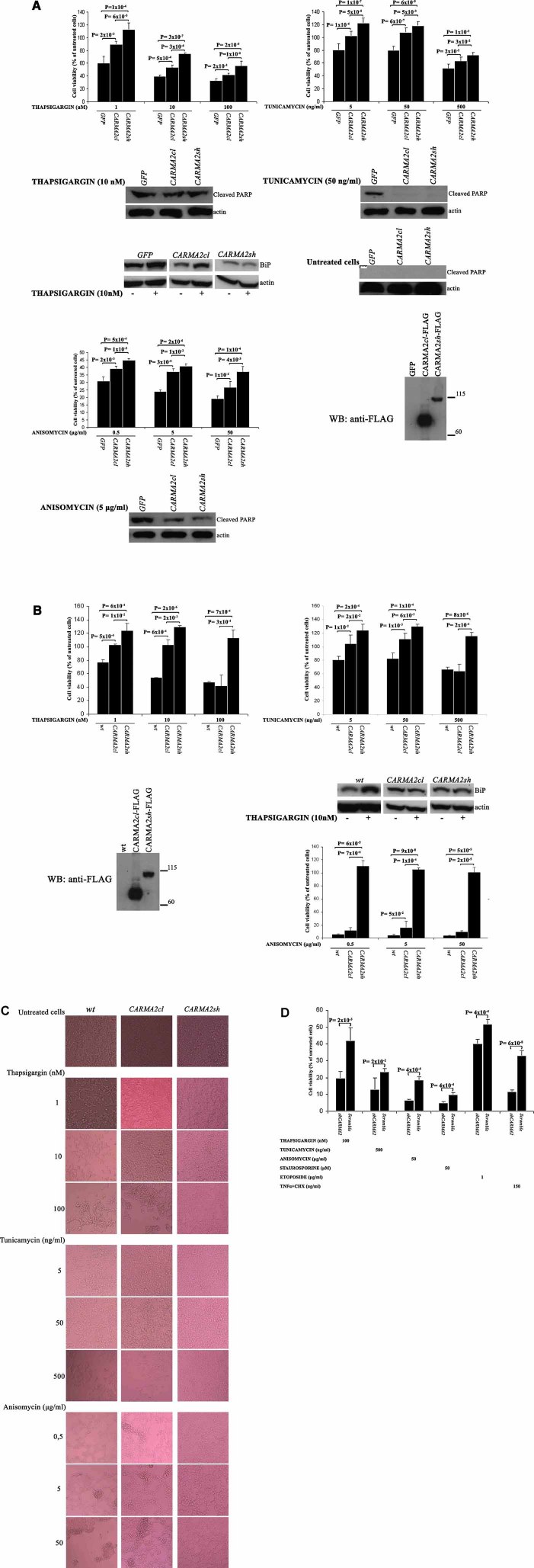
CARMA2*sh* expression protects cells from apoptosis. A: HEK-293 cells infected with a lentiviral vector expressing the indicated cDNAs were exposed to the indicated drugs for 24 h and then analyzed for apoptosis using ATPLite™ kit analysis. Each data point represents the mean ± SD cell survival expressed as percentage of untreated cells in six replicates. Statistical analysis was by the one-tailed unpaired Student's *t*-test. Bottom parts: Immunoblots analysis of expressed FLAG-tagged CARMA2*sh*, CARMA2*cl* in untreated cells, and cleaved PARP or BiP upon exposure to the drug. B: HeLa cells, stably transfected with an expression vector encoding for the indicated polypeptides, were treated and analyzed as in (A). Each data point represents the mean ± SD cell survival expressed as percentage of untreated cells in six replicates. Statistical analysis was by the one-tailed unpaired Student's *t*-test. C: Phase contrast micrographs (20×) of HeLa cells stably expressing the indicated polypeptides and exposed for 24 h to the indicated drugs. D: HeLa cells infected with a lentiviral vectors expressing a shRNAs targeting CARMA2 were exposed to the indicated drugs for 24 h. Extent of cell death was assessed using the ATPLite™ kit. Each data point represents the mean ± SD cell survival expressed as percentage of untreated cells in six replicates. Statistical analysis was by the one-tailed unpaired Student's *t*-test.

Since TRAF2 regulates apoptotic signal transduction pathways starting from membrane receptors and the ER membrane (Rothe et al., 1994; Yoneda et al., 2001; Mauro et al., 2006), we investigated the involvement of CARMA2*sh* in cell death induced by different stimuli. For this, we established HEK-293 cell lines that stably expressed FLAG-tagged CARMA2*sh* or CARMA2*cl* through a lentiviral expression system and then examined their responses to inducers of programmed cell death. The expression of ectopic CARMA2*sh* or CARMA2*cl* was assumed by immunoblot experiments; GFP is an irrelevant protein and here was used as a control. As shown in Figure 6A, expression of CARMA2*sh*, and partially that of CARMA2*cl*, confers resistance to apoptosis induced by thapsigargin, tunicamycin, and anisomycin treatment both in HEK-293 cells (Fig. 6A) and HeLa cells (Fig. 6B,C). Induction of ER stress was monitored by assessing the expression level of ER stress marker protein BiP (Dudek et al., 2009). At a lesser extent, CARMA2*sh*, but not CARMA2*cl*, protects cells also from death induced by etoposide, staurosporine, and TNFα treatment (data not shown).

To further assess the biological significance of CARMA2 in programmed cell death, CARMA2 expression was silenced by siRNA. We identified a siRNA which partially silenced CARMA2 expression in HeLa and HEK-293 cells (data not shown). When CARMA2 expression was silenced by this siRNA, apoptotic response was significantly increased compared to control siRNA (Fig. 6D). These results indicated that increased expression of CARMA2 has a protective role in apoptotic pathways.

## Discussion

In this work, we describe the identification and the functional characterization of two novel isoforms of CARMA2, namely CARMA2*sh* and CARMA2*cl*. The expression pattern of these newly identified transcripts is, however, different. In fact, while CARMA2*sh* appears to be expressed by the vast majority of cell lines analyzed, CARMA2*cl* appears to be expressed by few cell types (only 2 out of 14 cell lines analyzed). This suggests that the functions performed by the corresponding polypeptides might be different, namely CARMA2*sh* would play a biological function common to many cell types, whereas the function of CARMA2*cl* may be more cell-type specific. In addition, the two CARMA2 isoforms behave differently compared to CARMA2FL. In fact, while CARMA*sh*, similarly to CARMA2FL, retains the ability to activate NF-κB, CARMA2*cl* is unable to positively regulate this transcription factor.

The identification of these two alternative transcripts of CARMA2 is particularly intriguing for several aspects regarding the functional activity of CARMA2 and the CARMA proteins in a broader biological context. In fact, both CARMA2*cl* and CARMA2*sh* lack the SH3, PDZ, and GuK domains (MAGUK), which are essential for membrane localization and lipid raft recruitment of CARMA proteins (reviewed in Lin and Wang, 2004; Thome, 2004). This observation raises the interesting possibility that CARMA2*sh* and CARMA2*cl* may regulate signal transduction pathways of stimuli starting not from the cell membrane, but rather from intracellular compartments. Indeed, the most evident biological aspect that these two isoforms appear to affect is apoptosis induced by thapsigargin, tunicamycin, and anisomycin treatment. Both thapsigargin and tunicamycin induce ER stress by inhibiting ER-resident Ca2-ATPase, and N-glycosylation, respectively. Anisomycin is a translational inhibitor and strongly activates the stress-activated mitogen-activated protein (MAP) kinases pathway. Thus, altogether these observations are consistent with the hypothesis that these CARMA2 isoforms may regulate intracellular signals, particularly those arising from the ER. In this context, the finding that the functional activity of CARMA2*sh* relies on TRAF2 is undoubtedly relevant, since TRAF2 is involved in signaling from ER being able to interact with Ire1 (Urano et al., 2000), one of the ER transmembrane proteins involved in initiating signals from the ER. In addition, TRAF2 mediates activation of both the JNK/SAPK and the NF-κB pathways following ER stress (Urano et al., 2000; Leonardi et al., 2002; Mauro et al., 2006). Therefore, we propose that while CARMA2FL similarly to CARMA1 and CARMA3 may transduce stimuli starting from the cell membrane, CARMA*sh* and CARMA*cl* transduce stimuli starting from intracellular compartments.

The mechanism through which CARMA2*sh* protects cells from programmed cell death could obviously be related to the ability of this protein to induce activation of NF-κB transcription factor, which is well known to regulate transcription of anti-apoptotic genes.

However, it is evident that other protective mechanisms may exist, since even CARMA2*cl* partially protects from induced apoptosis, although unable to activate NF-κB. Thus, one can assume that different CARMA2 isoforms might likely impact other functions of this protein, in addition to regulation of apoptosis and NF-κB activation. In conclusion, our study revealed the existence of alternative splice variants of CARMA2, suggesting that the presence of distinct combinations of CARMA2 splice variants might potentially affect its biological activity. Clearly, there are many interesting question concerning CARMA2 function that require further investigation. In this context, the generation of animal models genetically modified in the locus encoding for CARMA2 will be certainly of enormous value to shed some light on the physiological role of this protein.

## Materials and Methods

### Reagents

Sources of antibodies and reagents were the following: anti-FLAG, anti-HA Sigma (St. Louis, MO); anti-TRAF2, anti-BiP, anti-cleaved PARP Santa Cruz Biotechnology (Santa Cruz, CA); anti-BCL-10 antibody has been generated in our laboratory and has been described elsewhere (Guiet and Vito, 2000). Thapsigargin, tunicamycin, anisomycin, staurosporine, etoposide, and human recombinant TNFα were obtained from Sigma. ATPLite™ kit was purchased from PerkinElmer Corp. (Waltham, MA) and used according to the manufacturer's indication.

The siRNA (*Mission*) directed to CARMA2 was from Sigma.

Human cDNA (*Marathon-Ready-cDNA*) were obtained from Clontech (Mountain View, CA).

### Cell culture and transfection

HEK293 and HeLa cells were maintained in Dulbecco's modified Eagle's medium (DMEM) supplemented with 10% FBS. DNA plasmids were transfected into cultured cells by calcium–phosphate methods or using Lipofectamine 2000 (Invitrogen, Carlsbad, CA) according to the manufacturer's protocol. Jurkat cells were maintained in RPMI medium supplemented with 10% FBS. Retroviral infections were performed as previously described (Guiet et al., 2002).

### RT-PCR

Total RNA was isolated from cells or tissues using TRIzol reagent (Invitrogen). Expand High Fidelity PCR system (Roche, Basel, Switzerland) was used to amplify each of the CARMA2 splice variants. The reverse transcriptase reaction was performed using 1 µg of total RNA in a 20 µl reaction and 1 µl of the resulting cDNA was used in the subsequent amplification step along with 300 nM of each primer. To eliminate the possibility of genomic DNA contamination, RNA preparation was treated with RNase-free DNase. Amplification consisted of 42 cycles of 95°C for 30 s, 58°C for 25 s, 72°C for 40 s, and a final amplification step at 72°C for 5 min. The following oligos were utilized to amplify CARMA2FL: fwd: 5′-GCCTGGCTCCTGACGG-3′; rev: 5′-TCGGGGGCTCTGCTCC-3′. The following oligos were utilized to amplify CARMA2*sh*: fwd: 5′-caaagtggcgacctcggg-3′; rev: 5′-gcaatatataaagcatccatcac-3′. The following oligos were utilized to amplify CARMA2*cl*: fwd 5′-TGTCCCGCGTTCCCAGCTG-3′; rev: 5′-CTCCCGCAGCGAGTGGATG-3′.

### Generation of *CARMA2* polyclonal antibody

A DNA fragment encoding the fragment 108–497 of CARMA2FL was cloned into pET-28a (Novagen, San Diego, CA) to create an N-terminal Histidine-tagged fusion protein. The His fusion protein was purified by affinity chromatography on Nichel–Sepharose beads. Cell lysate preparation and purification of recombinant proteins were performed as recommended by the resin manufacturer (Sigma). The purified polypeptide was used for the immunization of rabbits to obtain polyclonal antibodies (InCura srl, Cremona, Italy).

### ATPLite assay

A total of 8 × 10^3^ cells per well were cultured in flat-bottom 96-well plate in quadruplicates in 10% FBS/DMEM medium. Cell viability was determined using ATPLite™ (PerkinElmer Corp.) kit according to the manufacturer's instructions.

### Transfections and NF-κB activation assays

4 × 10^4^ HEK293 cells/well were plated in standard 24-well culture dishes and transfected with the reporter constructs pRSV-β-gal and pNF-κB-Luc plus the indicated expression plasmids using the calcium phosphate method. The total amount of transfected plasmid DNA was adjusted with pcDNA3 vector such that it was constant within each individual experiment. NF-κB activation was assessed by measuring luciferase activity (normalized for β-galactosidase expression) in cell extracts 16 h after transfection.

Wild-type (WT) and TRAF2^−/−^ murine embryonic fibroblasts (MEFs) were provided by Dr. T.W. Mak and Dr. W.C. Yeh (Yeh et al., 1997). For transfections of WT and TRAF2^−/−^ MEFs, 4 × 10^4^ cells/well were plated in 24-well culture 24 h prior to transfection. Expression and reporter plasmids were transfected using the Attractene (Qiagen, Milano, Italy). The cells were lysed and assayed 24 h after transfection using the luciferase assay system (Promega, Madison, WI) according to the manufacturer's instructions.

### Immunofluorescence

1 × 10^4^ HEK293 or HeLa cells were grown and transfected in chamber slides. Sixteen hours after transfection, cells were fixed in 4% paraformaldehyde for 15 min at room temperature and then permeabilized in PBS/0.1% Triton X-100. Cells were incubated for 30 min in 5% FCS–PBS with primary antibodies were, followed by several washes with 5% FCS–PBS, and then incubating for 30 min with secondary antibody in 5% FCS–PBS. All steps were done at room temperature.

### Immunoblot analysis and coprecipitation

Cell lysates were made in lysis buffer (150 mM NaCl, 20 mM Hepes, pH 7.4, 1% Triton X-100, 10% glycerol, and a mixture of protease inhibitors). Proteins were separated by SDS–PAGE, transferred onto nitrocellulose membrane, and incubated with primary antibodies followed by horseradish peroxidase-conjugated secondary antibodies (Amersham Biosciences, Piscataway, NJ). Blots were developed using the ECL system (Amersham Biosciences). For co-immunoprecipitation experiments, cells were lysed in lysis buffer and immunocomplexes were bound to protein A/G, resolved by SDS–PAGE, and analyzed by immunoblot assay.
